# A Retrospective Study to Assess Survival Post-adrenal Metastasectomy in Our Regional Endocrine Surgery Unit

**DOI:** 10.7759/cureus.95171

**Published:** 2025-10-22

**Authors:** Lewis Blenkinsop, Peter Truran, Jason Ramsingh

**Affiliations:** 1 Surgery, NHS Lead Employer Trust North East, Newcastle upon Tyne, GBR; 2 General Surgery, Royal Victoria Infirmary, Newcastle upon Tyne, GBR; 3 General Surgery, Newcastle Upon Tyne Hospitals NHS Foundation Trust, Newcastle upon Tyne, GBR

**Keywords:** adrenal, adrenalectomy, cancer, endocrine surgery, metastasectomy, metastasis, survival

## Abstract

Aim: The aim was to assess survival in patients post-adrenalectomy for metastasis from a non-adrenal primary.

Method: We reviewed our local database for all patients who underwent adrenalectomy for the indication of metastasis from January 2019 to December 2024 (27 cases). We then excluded patients with pathology results showing a non-metastatic lesion (four patients) and patients in whom adrenalectomy was unsuccessful (three patients). This left us with 20 patients who successfully underwent an adrenal metastasectomy. Eleven of the patients had primary lung tumors, four melanomas, two renal, two colon and one breast.

Results: Of the 20 cases the median age was 65.6 years, 13 patients were female and the median length of stay was one day. Ten operations were performed laparoscopically, six robotically and four open. The median tumor size was 3.5cm (1.6-12cm). There was one reported complication of intraoperative bleeding but the patient did not require a blood transfusion. Twelve patients went on to have further systemic treatment postoperatively.

A Kaplan-Meier survival analysis was performed, resulting in a one-year overall survival (OS) of 86%, three-year of 52% and five-year of 43%. The overall survival and progression-free survival (PFS) were worse for patients with lung cancer primaries than other primaries (three-year OS 25% vs 64% three-year PFS 0% vs 67%).

Conclusion: Our unit's post-operative survival is in line with the currently available data around survival post-adrenal metastasectomy. Lung cancer primary, as an indication for surgery, was more prevalent than expected in this data set. This high prevalence, coupled with the difference in OS and PFS between lung and non-lung primary, is noteworthy and warrants further investigation.

## Introduction

There are a variety of indications for adrenalectomy, including primary aldosteronism, pheochromocytoma and adrenocortical carcinoma [1]. Between January 2019 and December 2024 our endocrine surgery unit performed 282 adrenalectomies, the vast majority of which were for those indications listed previously. However, 27 of these cases were performed due to a presumed metastasis being present in the adrenal. Surgery for these patients would be considered when the patient is referred from the multidisciplinary team (MDT) responsible for the primary tumor to the endocrine surgery team. These patients would have either oligometastatic disease, disease that has spread to only a limited number of sites (including the adrenal), or oligoprogressive disease, disease that has multiple metastases that are currently static/regressing, but the adrenal tumor is continuing to progress. The decision that operative management may be beneficial is made by the MDT responsible for the primary tumor. The patient is then reviewed in the surgical clinic to discuss their fitness for theatre and preferences around treatment.

As with any operation, it would be important to counsel the patient on their alternative options, the main one being stereotactic body radiation therapy (SBRT). A meta-analysis conducted in 2020 by Chen et al. [2] found that one- and two-year overall survival (OS) after SBRT for isolated adrenal metastasis was 66% and 42% respectively. Local control in these cases was 82% and 63%. A similar systematic review by Liao et al. 2023 [3] found slightly better results with a one-year OS of 78.7% and a two-year OS of 62.9%. There are obviously differences in patient groups between those who undergo SBRT and those who undergo adrenalectomy as patients likely require a higher functional status for operative management. There does not appear to be any documented randomized controlled trial (RCT) comparing the two treatments and direct comparison between this data for SBRT and data for adrenalectomy will be skewed by the patient groups selected for the two treatment modalities. 

The most recent meta-analysis looking at adrenalectomy for secondary adrenal tumors was conducted by J Kong et al. [4] this consisted of 26 studies and 2279 patients. It found a one-year OS of 79.7%, a three-year OS of 49.1% and a five-year OS of 37.9%. 

Adrenalectomies themselves tend to have a low complication rate [5] and the operative risk is comparable to that of a laparoscopic cholecystectomy [6]. Operative modalities have generally moved towards a minimally invasive approach, but some larger tumors may require an open operation. Recently, there has been a movement towards early discharge following adrenalectomy. This includes the advent of day of surgery discharge which has been found to be safe in robotic cases [7]. Studies have also demonstrated the importance of experienced adrenal surgeons performing the operation to minimize complications [8], in our center there are two surgeons performing around 20 adrenalectomies each per year. 

The aim of this study is to assess our center's survival rates for patients who have undergone adrenalectomy for metastasis to the adrenal from a non-adrenal primary. Once we have calculated these local survival rates, we will then compare them to accepted published survival standards of operative and non-operative management. 

Abstract presented as a poster at BAETS 2025 annual meeting 2nd-3rd of October 2025.

## Materials and methods

We reviewed our local adrenalectomy database for all patients who underwent adrenalectomy for the indication of metastasis from January 2019 to December 2024 (27 cases). This database is constructed using the pooled surgeons' diaries for all adrenalectomies that take place in our unit and the pathology results are updated once returned meaning all adrenals removed due to metastasis would be included. Patients are considered for metastasectomy following referral from the MDT responsible for their primary cancer, they are then reviewed in surgical clinic where their fitness for an operation and their own wishes regarding treatment are discussed.

We then excluded patients who had the indication for surgery as metastasis but subsequent pathology results showing a non-metastatic adrenal lesion (four patients) and patients in whom adrenalectomy was unsuccessful (three patients). This left us with 20 patients who successfully underwent an adrenal metastectomy for a non-adrenal primary. 

We then calculated a Kaplan-Meier survival curve for both OS and progression-free survival (PFS). This was performed using SPSS version 29 statistical analysis software (IBM Corp., Armonk, NY, USA). OS was determined by accessing the patient's hospital and general practice records for any confirmation of death or documentation of their body being transferred to a mortuary. PFS was calculated based on the next cross-sectional imaging that showed progression or size increase of any known tumor location or development of new metastasis according to the formal radiology report. In one instance a patient's pre-operative scan was reviewed again due to the development of neurological symptoms seven days postoperatively. At this review it was found to contain metastases that were not originally reported and would have precluded surgery; their PFS was listed as one day. 

We then went on to separate data based on primary location to calculate OS and PFS for lung cancer primary vs other primary. 

Finally we reviewed oncology letters to see if patients were given subsequent systemic treatment for their primary cancer. 

## Results

A total of 20 cases were included in the study with the median age being 65.6 years; there were 13 females and the median length of stay was one day (one to six days). Ten operations were performed laparoscopically, six robotically and four open. The median tumor size was 3.5cm (1.6-12cm). The choice of operative approach was dependent on surgeon preference, not necessarily size of tumor. There was one reported complication of intraoperative bleeding, but the patient did not require a blood transfusion and was able to be discharged on postoperative day one. Twelve patients went on to have further systemic treatment postoperatively; five of these were lung cancer primaries who received either carboplatin, pembrolizumab or a combination of both. 

Two of these 20 cases were for oligo-progressive disease, one was a lung primary and one was a melanoma. The other 18 were performed for oligometastatic disease. 

In six cases the metastases were discovered at the time of primary cancer diagnosis or at initial staging of their primary cancer, four of these were lung primaries, one was melanoma and one was renal. The other 14 had their diagnosis after beginning treatment for their primary tumor or after this treatment was completed and it was discovered in post-treatment monitoring scans. 

The number of cases performed each year varied as represented in Table 1 below. Included in this table are the outliers who had their operation abandoned due to the tumor being unresectable and patients who had the tumor resected but subsequently had pathology results showing it to be benign. In all of the cases of failed resection the operation began laparoscopically; one required conversion to open intraoperatively. In the cases with negative pathology, two were performed laparoscopically and two were robotic-assisted. 

**Table 1:** Number of Resections by Year

Year	Total listed resections	Successful resection with positive pathology	Failed resection	Successful resection negative pathology
2024	9	8	0	1
2023	3	2	0	1
2022	4	1	2	1
2021	3	3	0	0
2020	2	2	0	0
2019	6	4	1	1

The breakdown of primary location is shown in the table below (Table 2). Lung cancer was the primary in 11/20 (55%) of cases. 

**Table 2:** Location of Primary Tumor

Primary Location	Number of Cases
Lung	11
Melanoma	4
Renal	2
Colon	2
Breast	1

**Figure 1:**
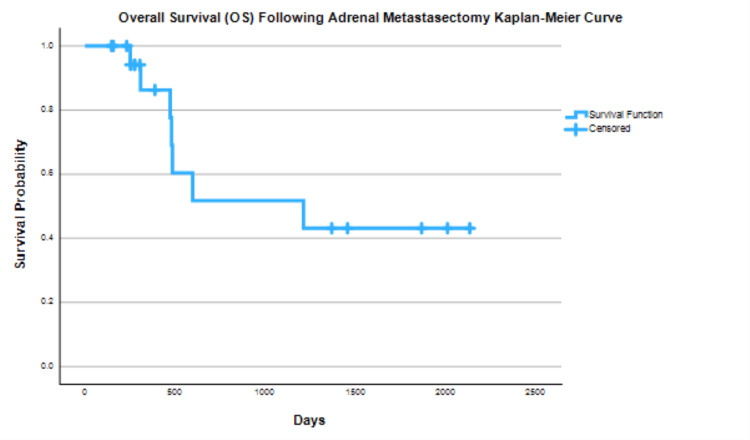
Overall Survival (OS) for all Primary Sources Post Adrenal Metastasectomy

A Kaplan-Meier survival curve analysis was performed on all 20 cases (Figure 1) resulting in a one-year OS of 86%, a three-year OS of 52% and a five-year OS of 43%. 

**Figure 2:**
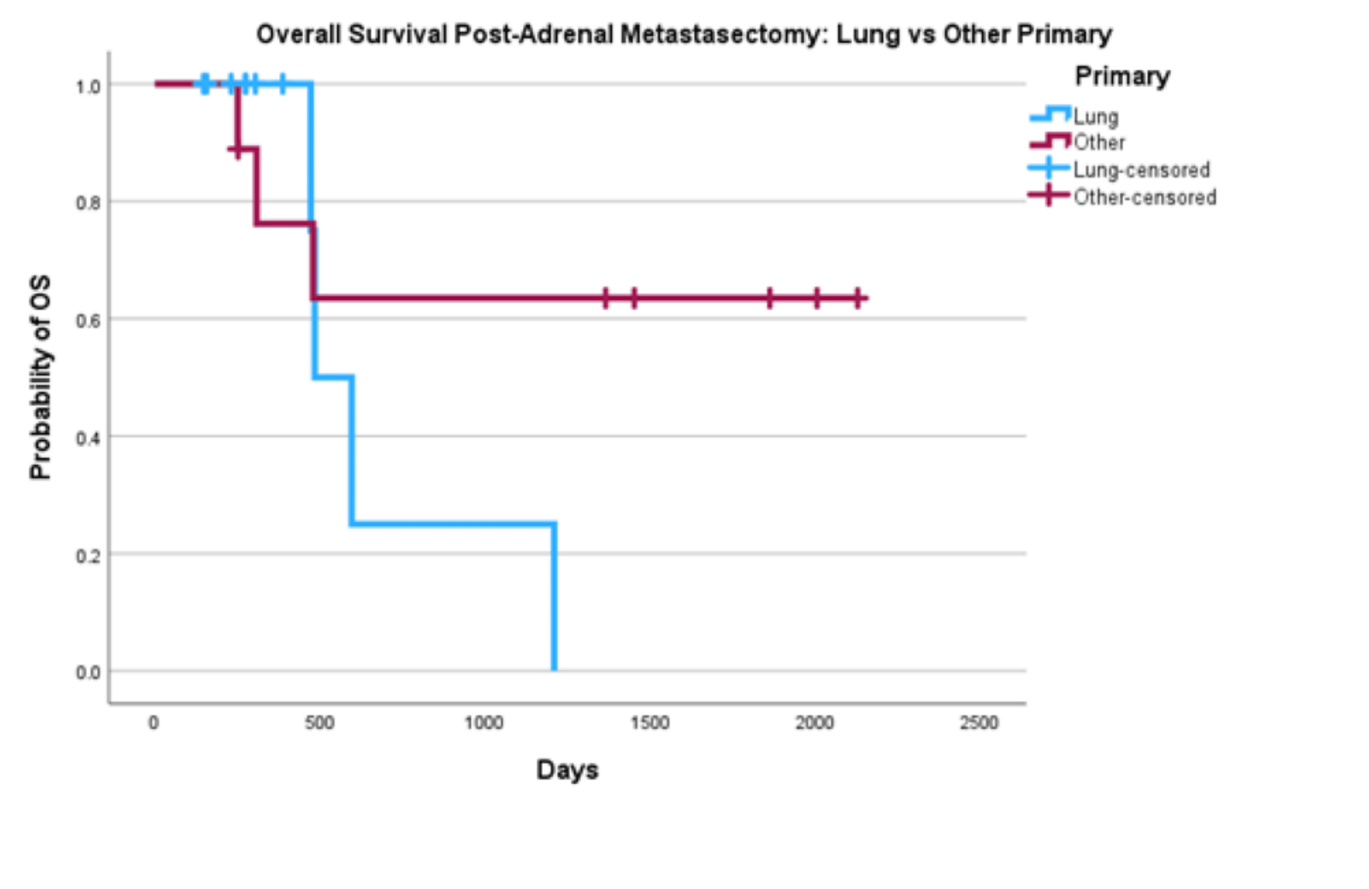
Overall Survival of Lung Primary vs Other Primaries Post Metastasectomy

This was then compared to OS for lung vs other primary (Figure 2). Lung cancer primaries had a one-year OS of 100% a three-year OS of 25% and a five-year OS of 0%. Other primaries had a one-year OS of 76%, a three-year OS of 64% and a five-year OS of 64%. 

**Figure 3:**
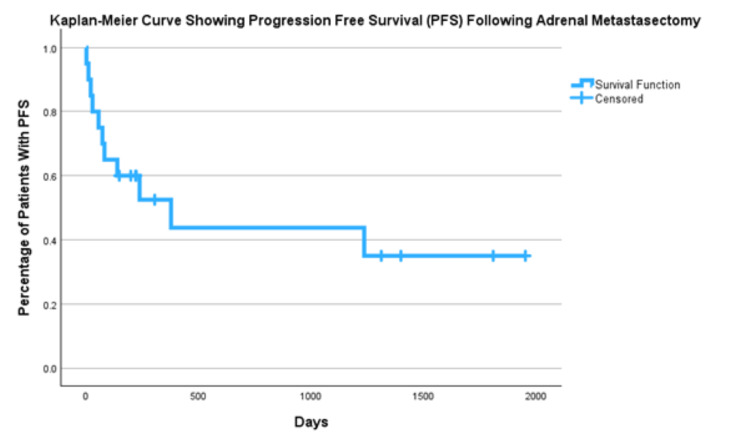
Progression Free Survival for All Primary Cancers Post Metastasectomy

**Figure 4:**
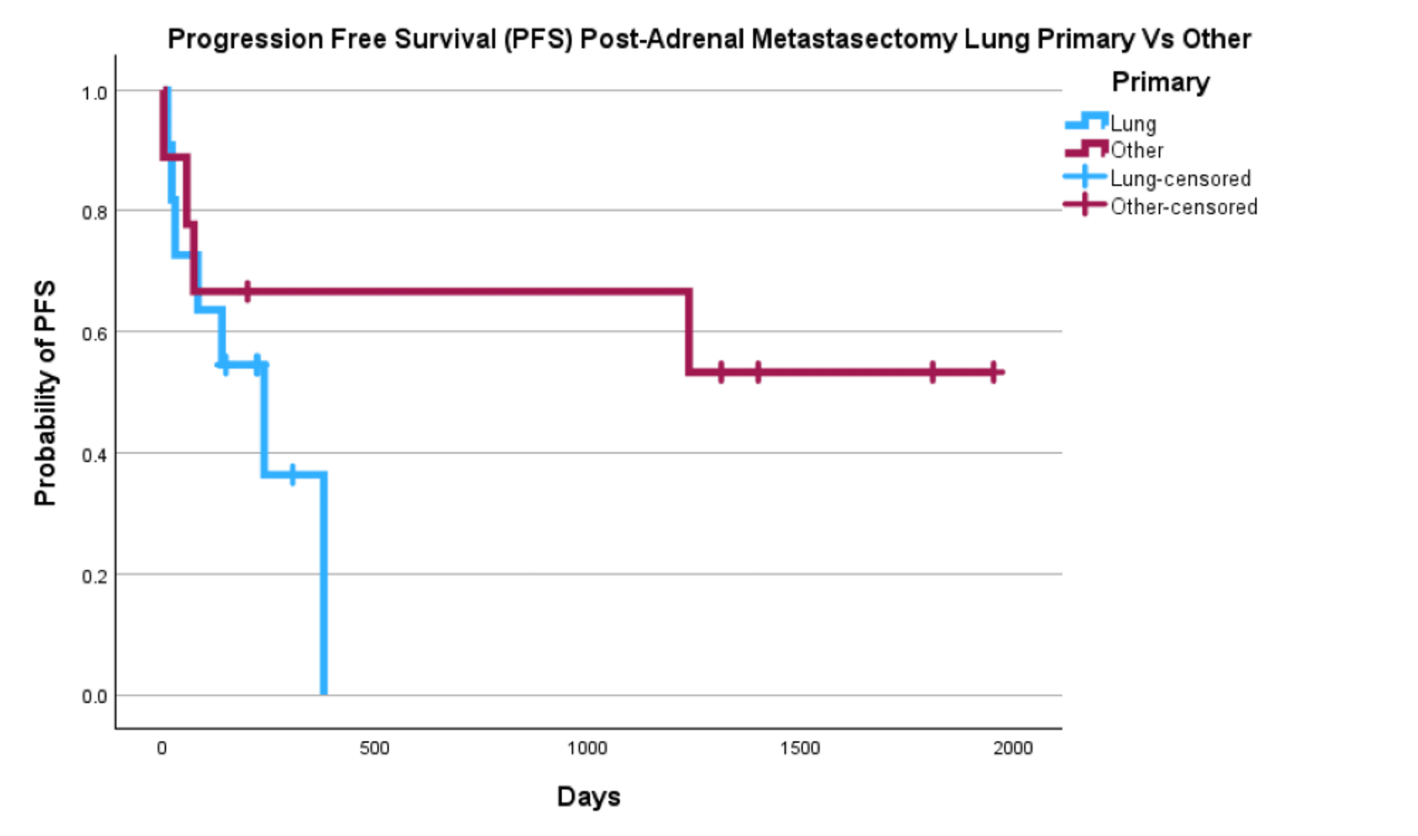
Progression Free Survival of Lung vs Other Primary Post Metastasectomy

PFS for all primaries was 53% at one year, 44% at three years and 35% at five years (Figure 3). We then compared PFS for lung primaries vs other primary (Figure 4) with results of one-year PFS 36% vs 67%, three-year 0% vs 67%, and five-year 0% vs 53%.

The survival findings are summarized below (Table 3). 

**Table 3:** Survival by Primary Location Post Metastasectomy OS: overall survival, PFS: progression-free survival

Primary location	One Year OS	Three Year OS	Five Year OS	One Year PFS	Three Year PFS	Five Year PFS
All	86%	52%	43%	53%	44%	35%
Lung	100%	25%	0%	36%	0%	0%
Non-Lung	76%	64%	64%	67%	67%	53%

## Discussion

This is a relatively small case series of patients who underwent adrenalectomy for secondary adrenal tumors. The findings however of OS are in line with those of larger meta-analyses [4]. This data set can provide useful information for further meta-analyses and is helpful in highlighting the inferior outcomes of patients with lung cancer primaries. 

The finding that patients with lung primaries have worse outcomes when compared to other primaries is also in agreement with other larger studies. However our outcomes are worse than those found in the metanalysis which reported “The one-year, three-year, and five-year OS rates were 73.3 % (95 % CI: 65.6 %-83.1 %), 42.4 % (95 % CI: 32.9 %-54.6 %), and 35.9 % (95 % CI: 26.6 %-48.5 %)” [4]; in fact our outcomes fall outside the 95% confidence interval for all three measured time intervals with our one-year outcomes superior to those reported in the metanalysis and the three-year and five-year outcomes being inferior. This is likely a sign of the small sample sizes we were dealing with in this study. Of our 11 patients who had lung primaries, only two had their operation more than three years ago, both are now deceased, but one survived for 1209 days post-operatively. 

Eleven (55%) of our cases had a lung primary that had metastasized to the adrenal. In the literature it is interesting to see the distribution of primary cancer. In the 2024 meta-analysis while lung was the most common primary, it made up only 29.7% of the total [4]. Of the studies included in the analysis, the variety of which primary site was most common is vast and appears to be center-dependent. In those centers where adrenalectomies are performed by urologists they have a higher percentage of renal primaries including one paper that consisted of 1,635 adrenalectomies, all for a renal primary [9]. Another paper published in a urological journal lists the percentage of renal primary as 47.7% (50/106) compared to 32.3% (34/106) lung [10]. In comparison, studies by endocrine surgeons have shown a larger spread of primary location. A European multicenter study found lung cancer to be the most common primary with 46.7% of cases (148/317 total cases) [11]. Our data highlights one of the benefits of a dedicated endocrine surgery team; by accepting referrals for adrenalectomy from the team responsible for the primary tumor we are able to offer our service to a wider range of patients, not just those with renal primaries. However, our over-representation of lung primaries may be due to the fact that adrenalectomies for these patients become a well-known option within the respiratory department, leading to us receiving more referrals and perhaps operating on more patients with this condition than would be representative in the global population. This may also explain why our three- and five-year OS for lung cancer is inferior to those widely published as we operate on more comorbid/advanced patients in this group. This also suggests an area of development for our unit as it is likely worthwhile to offer adrenalectomies to more patients with non-lung primaries given how they are underrepresented in this data. 

Reviewing the data it is interesting to see the shift towards minimally invasive surgery with only four of our cases being performed open and also the availability of experienced endocrine surgeons to offer shorter stays in hospital. Our data showed a low complication rate (one in 20) and a short median hospital stay (one day). Reviewing papers from the early 2000s, minimally invasive rates were much lower; one paper had only one out of 20 cases performed laparoscopically [12]. While this is an expected finding, there can be no doubt that the shift to minimally invasive surgery and short inpatient stays after operative intervention [7] will make surgery for adrenal metastasis a more attractive option. 

In the introduction to this article, we discussed the option of SBRT for adrenal metastasis, and we touched on how comparison between those patients suitable for surgery and those who undergo SBRT is limited due to SBRT being offered in more advanced disease and to more co-morbid patients. With those same caveats in place, a few historical papers compared operative to non-operative management. These were not randomized trials but retrospective studies, so their value is limited. One paper compared outcomes in metastatic lung cancer and found that those undergoing surgery had a five-year survival of 34% compared to 0% in those on chemotherapy alone [12]. Another paper found that median survival with surgery and chemotherapy was 31 months compared to 8.5 months with chemotherapy alone [13]. This poses a challenge as not only has the operative approach to adrenalectomy changed with time so too have the chemotherapy and immunotherapy agents available. It is likely that these studies from 1996 and 2011 would have had better outcomes with the oncological treatments available today and the difference in survival would not have been as stark. This can be demonstrated with cancer mortality rates falling by 37% in men between 1993 and 2018 [14] and lung cancer mortality specifically falling by 40% in men between 1990 and 2019 [15]. As we see adrenalectomy as a reasonable treatment choice, it is unlikely we will ever be able to compare outcomes fairly between operative and non-operative approaches to the management of isolated metastasis given the ethical considerations of any prospective trials.

The limitations of this study have been briefly touched upon already, namely the limited study size. This is also a single-center retrospective study, and as mentioned, there will be significant challenges to any prospective work in this field. Three surgeons' data are included, although one retired midway through the data collection period. The surgeons are high-volume adrenal surgeons and their data can not necessarily be extrapolated to other centers.

## Conclusions

Our endocrine surgery unit has comparable overall survival for adrenalectomy in non-adrenal primaries to those found in the literature. We have excellent complication and length of stay data. 

A larger percentage of the metastasectomies that we perform are on patients with lung primaries when compared to the literature. We have associated poorer long-term outcomes in these patients and this is possibly due to us operating on more advanced or comorbid patients. An alternative explanation for this is that our limited long-term data on these patients do not provide adequate information on outcomes at greater than three-year follow-up. It would be important for us to repeat this data collection in five years to see if the outcomes change. 

It is likely that as adrenalectomies become a recognized option in oligometastatic disease we will have a higher workload in this field and our unit should encourage other departments to refer to us as appropriate. This coupled with advances in cancer care and pharmaceutical agents will likely see improved long-term outcomes in suitable patient groups.
